# The association between physical activity and mental health in medical postgraduates in China during COVID-19 pandemic

**DOI:** 10.3389/fpsyt.2022.1036414

**Published:** 2022-12-02

**Authors:** Cai-Ling Yue, Xing Ge, Man Liu, Beibei Zhang, Stephane Koda, Chao Yan

**Affiliations:** ^1^Personnel Division of Xuzhou Medical University, Xuzhou, China; ^2^Jiangsu Key Laboratory of Immunity and Metabolism, Jiangsu International Laboratory of Immunity and Metabolism, Xuzhou Laboratory of Infection and Immunity, Department of Pathogenic Biology and Immunology, Xuzhou Medical University, Xuzhou, China

**Keywords:** Chinese medical postgraduates, physical activity, negative emotion, self-efficacy, resilience

## Abstract

**Background:**

Chinese medical postgraduates (CMPs) are a special subpopulation that has a great risk of mental health due to high workload, and heavy academic and clinical pressure during the COVID-19 pandemic. Physical activity has been demonstrated to be positive for the mental health of human being. However, little is known about the risks to mental health among CMPs as well as the potential effects of physical activity on mental health in CMPs during the COVID-19 pandemic.

**Aims:**

In the present study, the aims are to (1) investigate the levels of PA and mental health that CMPs had; (2) to identify the potential factors that contribute to mental health among CMPs; (3) to explore the potential effects of PA on the mental health in CMPs.

**Methods:**

The survey was conducted online across 25 provinces of mainland China in 28 medical colleges or universities with structured questionnaires. Physical Activity Rating Scale-3 (PARS-3), Depression, Anxiety, Stress Scales (DASS-21), and General Self-Efficacy Scale (GSES) were employed to assess the levels of PA, Negative emotional states, and self-efficacy of the participants, respectively. Resilience was evaluated using Connor-Davidson Resilience Scale (CD-RISC). The chi-square and Wilcoxon rank-sum tests were used to compare inter-group differences in demographic data and mental health conditions. Spearman's rank correlation test and partial correlation analysis were used to assess associations between exercise scores and mental health.

**Results:**

We found some socio-demographic variables such as the location, education levels of parents and the levels of degrees they are training had potential effects on outcomes of mental health among 2,217 CMPs (*P* < 0.05); furthermore, we also found that PA was negatively correlated with the negative emotion (*r* = −0.045, *P* < 0.05) such as depression (*r* = −0.052, *P* < 0.05), anxiety and stress, (*r* = −0.051, *P* < 0.05) but positively correlated with the self-efficacy (*r* = 0.143, *P* < 0.001) and resilience (*r* = 0.192, *P* < 0.001) among Chinese postgraduate medical students.

**Conclusion:**

We concluded that for Chinese postgraduate medical students, taking part in physical activity would depress negative emotions such as depression, anxiety, and stress, but improve their self-efficacy and resilience, which will benefit them in completing their studies and training.

## Introduction

Medical postgraduate education is a critical stage of training high-qualified clinicians and will make a profound influence on the global healthcare system. Globally, the demands of healthcare service are gradually increasing due to the rapid population growth, an expanding aging population, and the COVID-19 pandemic, which requires more high-qualified clinicians (1, 2). The situation highlights the significance of Medical postgraduate education. Actually, the last 30 years have witnessed a dramatic increase number of Chinese medical postgraduate students (CMPs). According to the China Health Yearbook from National Healthcare Commission, there are more than 33 000 medical postgraduate students in China in the year 2020, compared with ~1 300 in the 1900s (3). These students are undergoing postgraduate specialty training and will become the main force of health care in the country in the coming decades. Despite this, there are still great demands in the labor force of health care, for example, there are only 1.8 physicians in every 1,000 people in China while 3.6 and 4.6 physicians serve 1,000 people in Austria and Norway, respectively (4). Furthermore, the heavy workload of medical postgraduate is also due to the higher requirements of theory and practical skills, compared to the students from other disciplines (5, 6).

China has a unique educational system for training clinicians after the students graduated from medical school for 5 years of learning (7). Different from other countries, the education of medical postgraduate in China is divided into two different levels: master's and doctorate, which is parallel with the training mode of the Ph.D. degree in the UK. Due to different purposes, there are two different types of medical postgraduate: one is postgraduate with an academic degree (PAD) and the other is postgraduate with a professional degree (PPD) (8, 9). PAD is aimed to improve theoretical knowledge and academic performance, which means that it will take plenty of time to participate in biomedicine research in addition to clinical practice (10). In the contrast, PPD lays particular stress on the abilities of clinical practice, the goal of which is to train high-qualified clinicians with strong innovation capability (7).

Recently, Chinese government has reformed postgraduate medical education and standardized residency training (SRT), which requires PPD students to have SRT for at least 33 months while finishing postgraduate medical education (7, 11). For PAD students, also have high pressure to publish high-quality papers as one of the requirements for obtaining degrees in many institutions (9). Therefore, CMPs, regardless of PAD or PPD, as the backbone working in the Chinese healthcare frontline, is currently experiencing a high workload and heavy academic or clinical pressure. Because heavy clinical and academic workloads can cause tension, which may bring great risks of mental health problems (such as depression, anxiety, and stress) and poor self-efficacy. There was more than 30% of medical postgraduate students have mild to severe depression symptoms according to a national cross-sectional study in China (12). In another cross-sectional study with a small, specific population (~500 participants of dental postgraduates), more than 30% of Chinese dental postgraduates suffered from job burnout, career choice regret, and depressive symptoms in a cross-sectional study (13). In a comparative study, Peng et al. (14) found that both medical and non-medical postgraduates have mental distress but medical postgraduates have higher perceived stress due to an unsatisfied healthcare environment.

Furthermore, the pandemic of COVID-19 may aggravate this situation (12). For example, a study showed that more than 2/3 of medical students had deteriorated mental health such as depression and anxiety during COVID-19 pandemic, which was higher than the general population (15). In China, there was also a study also showed more than half of medical postgraduates had mild anxiety symptoms during the COVID-19 pandemic (16). Because CMPs represent a large group in the system of medical care, the mental health of these students is an extensive concern in universities and hospitals. This problem can persist if not treated resulting in numerous undesirable personal and professional consequences (17).

Many studies have demonstrated that physical activities (PA) can have a positive effect on the mental and physical health of human beings. Moderate-to-vigorous PA can effectively maintain body weight, reduce the risk of diseases, benefit brain health, and improve negative emotional states and mental symptoms, although the mechanisms may be different (18). Furthermore, lack of PA is one of the three leading causes of chronic disease and premature death (19). Although there are several data about PA on the mental health of undergraduate medical students in China, few data about the association between PA and the mental health of CMP are available. Given this background, the aims of the present study were to (1) investigate the levels of PA and mental health that CMPs had; (2) identify the potential factors that contribute to mental health among CMPs; (3) explore the potential effects of PA on the mental health in CMPs. In the present study, we investigated the association between PA and mental health in this population. Our finding in the present study will provide the fundamental information and suggestion for to improve mental health of CMPs which will benefit the reform of postgraduate education.

## Materials and methods

### Ethics approval and consent to participate

The main procedures of the present study were reviewed and approved by the Ethical Committee of the Xuzhou Medical Univeristy (XZMU-2020-ZK043).

### Participants and study design

The survey was carried out online across 25 provinces of mainland China in 28 medical colleges or universities from 20 October 2020 to 5 April 2021 with structured questionnaires (Supplementary Table 1). The online questionnaire was undergone *via* an online survey platform (www.wjx.cn) which was distributed to postgraduates by the administration of postgraduates in medical colleges or universities. Before beginning the questions about their demographic data, participants were informed about the objectives of the study and provided with an e-signature to indicate their informed consent. Their majors that were engaged were inquired about. A total of 2,595 Chinese postgraduate students volunteered to attend this study and 2,424 participants (93.41%) had valid responses to this survey. Of these participants, 2,217 students were in the major of medicine. In the following, these post-graduate students majoring in medicine were subjected to questionnaire demographic characteristics, including age, gender, major, location, degree applied for, and so on (Table 1), and further questions in the following.

**Table 1:** Descriptive statistics of the participants in the present study.

**Variable**		**Frequency**	**Percentage (%)**
Genders			
	Male	702	31.66
	Female	1,515	68.34
Ages			
	20–23	562	25.35
	24–27	1,388	62.61
	28–31	193	8.71
	32–35	57	2.57
	>36	17	0.77
Location			
	City	1,090	49.17
	Villages	1,127	50.83
Income of family			
	Low	294	13.26
	General	1,818	82.00
	High	105	4.74
Education of parents			
	High and above high school	869	39.20
	Senior	989	44.61
	Junior	335	15.11
	Unschooled	24	1.08
Degrees			
	Master	2,151	97.02
	Doctor	66	2.98
Types of degree			
	Academic	782	35.27
	Professional (clinical medicine, stomatology)	1,166	52.59
	Professional (others)	269	12.13
Grade			
	Level 1	1,201	54.17
	Level 2	671	30.27
	Level 3	345	15.56
Total		2,217	100

### Measurements

Physical Activity Rating Scale-3 (PARS-3) was employed to assess student PA level, which was revised by Liang (20). The scale mainly includes three items of physical exercise intensity (what is the intensity of PA that you usually engaged in?), duration (*How long do you spend in each PA session in 1 week?*), and frequency (*how often do you spend in each PA session in 1 week*). The Likert 5-scale scoring standard is adopted, and the corresponding scores are 1–5 points respectively. Physical exercise score = intensity score × (time score−1) × frequency score. The PA score interval ranged from 0 to 100 points. A higher score means more physical activity. The test-retest reliability of PARS-3 in this study was 0.82.

Negative emotional states were evaluated using Depression, Anxiety, Stress Scales (DASS-21) (21). DASS-21 includes three dimensions of stress, anxiety, and depression with 21 items and each dimension has seven items. For each item, the Likert 4-scale scoring standard (0–3) was employed to evaluate each item. DASS-21 score ranges from 0 to 63. The higher score suggests a higher level of depressive and anxiety symptoms and higher pre**s**sure on participants. As confirmed by Sahebi et al. (22) this scale has validity and reliability between 0.77 and 0.79. The internal consistency of this questionnaire was approved (Cronbach's alpha: 0.870–0.893) in this study.

General Self-Efficacy Scale (GSES) was used to assess the level of self-efficacy of each participant (23). The GSES includes 10 items and each item was measured on a 4-point Likert (1–4 scores). The total score of GSES ranges from 10 to 40. A higher score indicates a higher capacity for self-efficacy. The internal consistency of this questionnaire was determined (Cronbach's alpha: 0.892) in this study.

Resilience was assessed using Connor-Davidson Resilience Scale (CD-RISC) developed by Connor-Davidson and adopted by Yu over the past month (24, 25). The scale consists of 25 items within three dimensions including hardiness, strength, and optimism. The response to each item is based on a 5-point Likert scale (1–5): 1 (not true at all) to 5 (true nearly all of the time). Thus, the total score of resilience ranges from 1 to 125, with higher scores suggesting greater resilience. Cronbach's alpha of the ADRS in the present study was 0.71–0.786.

### Statistical analysis

The normality of variables was tested using the Shapiro-Wilk normality test and the KS normality test. Continuous variables were presented as means ± SD for normally distributed data and medians and interquartile ranges (1st quartile, 3rd quartile) for non-normally distributed data. The chi-square and Wilcoxon rank-sum tests were used to compare inter-group differences in demographic data and mental health conditions as appropriate. Spearman's rank correlation test and partial correlation analysis were used to assess associations between exercise scores and mental health. *P* values less than 0.05 were considered statistically significant. SPSS 19.0 software (IBM, Armonk, NY, USA) was used for data analysis.

## Results

### Socio-demographic characteristics, PA, and mental health condition

Tables 1, 2 showed the key statistical data on the variables. there were a total of 2,217 CMPs attending the survey. Table 1 showed that male students constituted 31.7% of the sample and the percentage of female students in the total population was 68.3% in this survey. The participants aged 24–27 years old were the most subpopulation (62.6%). More detailed information including the location, income of the family, education of parents, degrees, and so on were shown in Table 1. In Table 2, the average score of PA in CMPs that took part was 14.37 (95% CI: 13.670–15.069, SD: 16.7953) assessed by PARS-3. The mean score of negative emotional states (the sum of Depression, Anxiety, and Stress assessed by DASS-21) was 8.147 (95% CI: 7.755–8.539, SD: 9.4225). Each variable that indicates mental health were also shown in Table 2.

**Table 2:** Descriptive analysis of PA and mental health index.

**Variable**	**Mean**	**95% CI–**	**95% CI+**	**SD**
Physical activity (PA)	14.37	13.67	15.07	16.80
Negative emotion	8.15	7.76	8.54	9.42
Depression	2.86	2.72	2.99	3.18
Anxiety	3.49	3.32	3.65	3.94
Stress	1.81	1.68	1.93	3.06
Self-efficacy	26.30	26.11	26.49	4.53
Resilience	85.35	84.70	85.99	15.49

### The potential factors on the mental health of CMPs

Next, we investigated the possible risk factors that can make effects the mental health of CMPs. As shown in Table 3, among these socio-demographic variables, we found that genders, ages, income, and types of degrees (academic or professional) have no significant effects on the mental health of CMPs. However, we found that the locations (villages or cities, χ^2^ = 10.369, *P* < 0.05)) and education levels of parents (above high school, senior, junior or illiteracy, χ^2^ = 7.250, *P* < 0.05) had effects on self-efficacy of CMPs. Furthermore, we also found that the levels of degrees (master's or doctorate) that they are pursuing had significant influences on the negative emotion including depression (χ^2^ = 8.172, *P* < 0.05), anxiety (χ^2^ = 6.708, *P* < 0.05), and stress (χ^2^ = 6.4, *P* < 0.05).

**Table 3:** The variables that possibly affect mental health among Chinese postgraduate students.

**Variable**	**Negative emotion**		**Depression**		**Anxiety**		**Stress**		**Self-efficacy**		**Resilience**	
	**χ^2^**	***P*-value**	**χ^2^**	***P*-value**	**χ^2^**	***P*-value**	**χ^2^**	***P*-value**	**χ^2^**	***P*-value**	**χ^2^**	***P*-value**
Gender	2.93	0.57	5.06	0.28	1.04	0.90	4.57	0.33	1.18	0.88	4.23	0.38
Ages	0.45	0.50	0.12	0.73	0.65	0.42	0.84	0.36	3.08	0.08	2.33	0.13
Location	2.86	0.41	2.89	0.41	2.41	0.49	2.32	0.51	10.37	0.02	5.77	0.12
Education of parents	1.80	0.41	2.35	0.31	1.67	0.43	0.24	0.89	7.25	0.03	0.93	0.63
Income	0.56	0.45	0.08	0.90	1.22	0.27	1.93	0.17	0.521	0.47	0.19	0.66
Levels of degrees	8.17	0.02	6.71	0.04	8.41	0.02	6.40	0.04	3.55	0.17	5.57	0.06
Types of degree	1.69	0.43	1.42	0.49	3.11	0.21	2.48	0.29	2.14	0.34	3.17	0.21

### The association between PA and mental health condition of CMPs

We further used Spearman's rank correlation test and partial correlation analysis to explore the association between PA and the mental health conditions of CMPs (Table 4). For PA, the data showed that there was a negative correlation between PA and negative emotions of students (*r* = −0.045, *P* < 0.05), between PA and Depression (*r* = −0.052, *P* < 0.05), between PA and stress (*r* = −0.051, *P* < 0.05). Furthermore, we also found that there was also a positive correlation between PA and self-efficacy (*r* = 0.143, *P* < 0.001), and between PA and Resilience (*r* = 0.192, *P* < 0.001). Specifically, we found that physical activity frequency was also negatively correlated with negative emotion (*r* = −0.045, *P* < 0.05), but was a positive correlation with self-efficacy (*r* = 0.063, *P* < 0.01) or Resilience (*r* = 0.096, *P* < 0.001); Physical activity intensity was also found to be positively correlated with Self-efficacy (*r* = 0.11, *P* < 0.001) or Resilience (*r* = 0.136, *P* < 0.001. For Physical activity duration, it was found that there was a negative correlation with scores of Negative emotion (*r* = −0.61, *P* < 0.01), but positively correlated with self-efficacy (*r* = 0.138, *P* < 0.001) or Resilience (*r* = 0.184, *P* < 0.001). Taken together, these data suggest PA has negative effects on negative emotions (such as Depression, Anxiety, and Stress), but positively improves self-efficacy and resilience in the group of CMPs.

**Table 4:** The relationship between physical activity on negative emotion, depression, anxiety, stress, self-efficacy, and resilience in medical postgraduate students in China.

**Variable**	**Negative emotion**		**Depression**		**Anxiety**		**Stress**		**Self-efficacy**		**Resilience**	
	***r***	***P*-value**	***r***	***P*-value**	***r***	***P*-value**	***r***	***P*-value**	***r***	***P*-value**	***r***	***P*-value**
Physical activity frequency	−0.05^*^	0.04	−0.03	0.12	−0.03	0.11	−0.05^*^	0.03	0.06^**^	0.003	0.1^**^	0.00
Physical activity intensity	0.004	0.87	−0.007	0.73	0.011	0.62	−0.01	0.80	0.11^**^	0.000	0.14^**^	0.00
Physical activity duration	−0.06^**^	0.004	−0.061^**^	0.004	−0.06^**^	0.005	−0.05^*^	0.01	0.14^**^	0.000	0.18^**^	0.00
Physical activity	−0.05^*^	0.02	−0.052^*^	0.01	−0.04	0.074	−0.05^*^	0.02	0.14^**^	0.000	0.19^**^	0.00

* *P* < 0.05.

** *P* < 0.01.

## Discussion

In the present study, we reported a comprehensive, national study of PA and mental health among postgraduate medical students in China. To our best knowledge, this study is the first report that investigated PA and mental health, as well as exploring their potential associations among postgraduate medical students in China. We found some socio-demographic variables such as the location, education levels of parents and the levels of degrees they are training had potential effects on outcomes of mental health among those Chinese postgraduate medical students; furthermore, we also found that PA was negatively correlated with the negative emotion such as depression, anxiety, and stress, but positively correlated with the self-efficacy and resilience among Chinese postgraduate medical students. Therefore, we concluded that for Chinese postgraduate medical students, taking part in physical activity would depress negative emotions such as depression, anxiety, and stress, but improve their self-efficacy and resilience, which will benefit them in completing their studies and training. Although CMPs have multiple pressures during accomplishing their studies and training, previous studies showed that there were no significant differences in the prevalence between medical students and non-medical students that have mental health problems, especially after the outbreak of COVID-19 (14, 26). Similarly, our study showed a moderated score of negative emotion in CMPs (Mean score: 8.17, 95% Cl: 7.755–8.539). The reasons that account for the phenomenon might be like this: as postgraduate medical students, they are equipped with the basic knowledge and skills to adjust their mental and physical conditions for adapting to the high pressures they have (23, 27), which were in line with our studies showing a relatively high resilience (mean score: 85.349) and self-efficacy (mean score: 28, full score is 40) among CMPs.

In our present study, we also found that levels of degrees (doctors or masters) had potential effects on mental distress, but the education levels of parents and the locations of CMPs exerted some influences on the self-efficacy of these students, which finally bring the effects on the academic achievements of GMPs. There is a study also showed that poor education of parents significantly affected an individual's schooling pursuits, which is in line with our present study (28). Because the requirements of obtaining degrees for master's and doctorate are quite different, for example, the doctor trainee is much higher heavier academic and clinical work to finish than those master trainee (11), the doctor candidates may be subjected to a higher pressure than that of the master trainee. Furthermore, there is a dual structure of urban and rural areas in China, which makes the urban-rural split very clear in economics, education, medical care, and so on (29). These differences may reflect that students have different tolerance to mental distress, self-efficacy, and resilience.

The relationship between PA and mental health has been well-documented in different populations of different ages and disease conditions (30, 31). There were many mechanisms by which PA improves mental health: at the molecular level, regular PA can increase the produce brain-derived neurotrophic factors (BNDF), which leads to the activation of ERK signaling pathway and inhibition of depressive-like behavior (32, 33). In addition, PA can change the structures and functions of the brain *via* the hypothalamic-pituitary-adrenal (HPA) axis, which is critical to meliorate the symptoms of depression and anxiety (34); at the psycho-social level, it is also suggested that psycho-social factors such as resilience were also involved in the PA-induced anti-mental disorders. In our present study, we found that PA especially the duration of PA had a negative relationship with negative emotions such as depression, anxiety, and stress, but self-efficacy or resilience among CMPs, which agreed with other studies although they surveyed different populations (20, 35, 36). These findings suggested that self-efficacy or resilience may probably be important mediators between PA and negative emotion (37). However, in our present study, we didn't any relationship between physical activity intensity and negative emotion, but it can be positively correlated with self-efficacy or resilience, suggesting that PA harmed negative emotion independent of the intensity of PA (38, 39).

In the present study, we investigated the potential effects of PA on the mental health of CMPs, a special population in China. Although we conducted a large investigation across 25 provinces in China, obtaining massive data, our study had some limitations: we didn't analyze the status of mental health that CMPs in detail. We will further analyze these data to find the potential risk factors that influence the mental health of CMPs. Additionally, since these mental disorders may have a potential effect on the academic performance of CMPs, which should be further investigated.

In summary, our study suggests physical activity would depress negative emotions such as depression, anxiety, and stress, but improve the self-efficacy and resilience of CMPs. Given the strong link between PA and mental health in medical postgraduate students in China, there are strong demands to urge CMPs to take part in physical exercise, which will not only benefit the postgraduate medical students themselves but also the whole medical care system.

## Data availability statement

The original contributions presented in the study are included in the article/Supplementary material, further inquiries can be directed to the corresponding author/s.

## Author contributions

C-LY and CY designed the experiments. C-LY collected the data. BZ and XG contributed to the data analysis. CY and SK wrote the paper. CY reviewed the final version of the manuscript and supervised the project. All authors read and approved the final version of the manuscript.

## Funding

This study was supported by the Project of Philosophy and Social Science Research in Colleges and Universities in Jiangsu Province (2021SJA1089). The funders had no role in the study design, data collection, analysis, decision to publish, or preparation of the manuscript.

## Conflict of interest

The authors declare that the research was conducted in the absence of any commercial or financial relationships that could be construed as a potential conflict of interest.

## Publisher's note

All claims expressed in this article are solely those of the authors and do not necessarily represent those of their affiliated organizations, or those of the publisher, the editors and the reviewers. Any product that may be evaluated in this article, or claim that may be made by its manufacturer, is not guaranteed or endorsed by the publisher.
