# Supplementary material for: The association between physical activity and mental health in medical postgraduates in China during COVID-19 pandemic
Source: Front Psychiatry. 2022 Dec 2;13:1036414. doi: 10.3389/fpsyt.2022.1036414 (PMC9755329; doi:10.3389/fpsyt.2022.1036414)
Supplement: Supplementary file 1 [file Table_1.docx]

| **Sampling site No** | **Sites (provinces, municipalities or autonomous regions)** | **Size** | **percents (%)** |
| --- | --- | --- | --- |
| 1 | Jiangsu | 780 | 35.18 |
| 2 | Jiangxi | 348 | 15.70 |
| 3 | Hebei | 201 | 9.07 |
| 4 | Inner Mongolia | 174 | 7.85 |
| 5 | Shandong | 173 | 7.80 |
| 6 | [Sichuan](javascript:openwindow('viewcity.aspx?activity=132754573&province=%u56db%u5ddd&sat=1',920,700)) | 173 | 7.80 |
| 7 | [Guangdong](javascript:openwindow('viewcity.aspx?activity=132754573&province=%u5e7f%u4e1c&sat=1',920,700)) | 87 | 3.92 |
| 8 | [Chongqing](javascript:openwindow('viewcity.aspx?activity=132754573&province=%u91cd%u5e86&sat=1',920,700)) | 77 | 3.47 |
| 9 | [Shannxi](javascript:openwindow('viewcity.aspx?activity=132754573&province=%u9655%u897f&sat=1',920,700)) | 46 | 2.07 |
| 10 | [Anhui](javascript:openwindow('viewcity.aspx?activity=132754573&province=%u5b89%u5fbd&sat=1',920,700)) | 35 | 1.58 |
| 11 | [Liaoning](javascript:openwindow('viewcity.aspx?activity=132754573&province=%u8fbd%u5b81&sat=1',920,700)) | 32 | 1.44 |
| 12 | [Henan](javascript:openwindow('viewcity.aspx?activity=132754573&province=%u6cb3%u5357&sat=1',920,700)) | 16 | 0.72 |
| 13 | [Beijing](javascript:openwindow('viewcity.aspx?activity=132754573&province=%u5317%u4eac&sat=1',920,700)) | 15 | 0.68 |
| 14 | [Guangxi](javascript:openwindow('viewcity.aspx?activity=132754573&province=%u5e7f%u897f&sat=1',920,700)) | 13 | 0.59 |
| 15 | [Shanghai](javascript:openwindow('viewcity.aspx?activity=132754573&province=%u4e0a%u6d77&sat=1',920,700)) | 13 | 0.59 |
| 16 | [Hubei](javascript:openwindow('viewcity.aspx?activity=132754573&province=%u6e56%u5317&sat=1',920,700)) | 12 | 0.54 |
| 17 | [Zhejiang](javascript:openwindow('viewcity.aspx?activity=132754573&province=%u6d59%u6c5f&sat=1',920,700)) | 6 | 0.27 |
| 18 | [Heilongjiang](javascript:openwindow('viewcity.aspx?activity=132754573&province=%u9ed1%u9f99%u6c5f&sat=1',920,700)) | 3 | 0.14 |
| 19 | [Shanxi](javascript:openwindow('viewcity.aspx?activity=132754573&province=%u5c71%u897f&sat=1',920,700)) | 3 | 0.14 |
| 20 | [Hunan](javascript:openwindow('viewcity.aspx?activity=132754573&province=%u6e56%u5357&sat=1',920,700)) | 2 | 0.09 |
| 21 | [Jilin](javascript:openwindow('viewcity.aspx?activity=132754573&province=%u5409%u6797&sat=1',920,700)) | 2 | 0.09 |
| 22 | [Fujian](javascript:openwindow('viewcity.aspx?activity=132754573&province=%u798f%u5efa&sat=1',920,700)) | 2 | 0.09 |
| 23 | [Tianjing](javascript:openwindow('viewcity.aspx?activity=132754573&province=%u5929%u6d25&sat=1',920,700)) | 2 | 0.09 |
| 24 | [Yunnan](javascript:openwindow('viewcity.aspx?activity=132754573&province=%u4e91%u5357&sat=1',920,700)) | 1 | 0.05 |
| 25 | [Ningxia](javascript:openwindow('viewcity.aspx?activity=132754573&province=%u5b81%u590f&sat=1',920,700)) | 1 | 0.05 |
| Total | | 2217 | 100 |

**Supplementary table 1 the sampling size and sources in the present study**
